# Diversity and roles of (t)RNA ligases

**DOI:** 10.1007/s00018-012-0944-2

**Published:** 2012-03-17

**Authors:** Johannes Popow, Alexander Schleiffer, Javier Martinez

**Affiliations:** 1 Institute of Molecular Biotechnology of the Austrian Academy of Sciences (IMBA), Dr. Bohrgasse 3, 1030 Vienna, Austria; 2 Bioinformatics Department, Research Institute of Molecular Pathology (IMP)–IMBA, Dr. Bohrgasse 7, 1030 Vienna, Austria

**Keywords:** RNA ligase, tRNA, 2′,3′-cyclic phosphate, *Sc*TRL1, Rnl1, Rnl2, HSPC117, DDX1, CGI-99, FAM98, ASW, RtcA, RtcB

## Abstract

The discovery of discontiguous tRNA genes triggered studies dissecting the process of tRNA splicing. As a result, we have gained detailed mechanistic knowledge on enzymatic removal of tRNA introns catalyzed by endonuclease and ligase proteins. In addition to the elucidation of tRNA processing, these studies facilitated the discovery of additional functions of RNA ligases such as RNA repair and non-conventional mRNA splicing events. Recently, the identification of a new type of RNA ligases in bacteria, archaea, and humans closed a long-standing gap in the field of tRNA processing. This review summarizes past and recent findings in the field of tRNA splicing with a focus on RNA ligation as it preferentially occurs in archaea and humans. In addition to providing an integrated view of the types and phyletic distribution of RNA ligase proteins known to date, this survey also aims at highlighting known and potential accessory biological functions of RNA ligases.

**Electronic supplementary material:**

The online version of this article (doi:10.1007/s00018-012-0944-2) contains supplementary material, which is available to authorized users.

## Introns within tRNA genes

Transfer RNAs (tRNA) are transcribed as precursor transcripts and are subjected to a series of posttranscriptional processing events before they are matured to fulfill their biological functions [1, 2]. Sequence analysis of tRNA genes in yeast [3, 4], and later in archaea [5], plants [6], and mammals [7], revealed the existence of tRNA genes disrupted by intervening sequences. Intron harboring tRNA genes are now known to occur in the genomes of organisms from all three domains of life [8]. After the discovery of intron-containing tRNAs [3–7], the mechanistic features of tRNA splicing were extensively studied [2, 9–11]. This review aims at providing an overview of the mechanistic aspects and enzymes involved in tRNA splicing across the three major lines of descent considering in particular a novel type of RNA ligases recently identified in bacteria, archaea, and humans.

## tRNA introns in bacteria, archaea, and eukaryotes

In contrast to bacterial pre-tRNAs—which are disrupted by self-splicing group I introns [12, 13]—archaeal and eukaryal pre-tRNA transcripts undergo enzymatic splicing. The latter achieves intron removal by endoribonucleolytic cleavage and subsequent ligation rather than by two consecutive transesterification events as employed by self-splicing introns or the spliceosome.

**Fig. 1:**
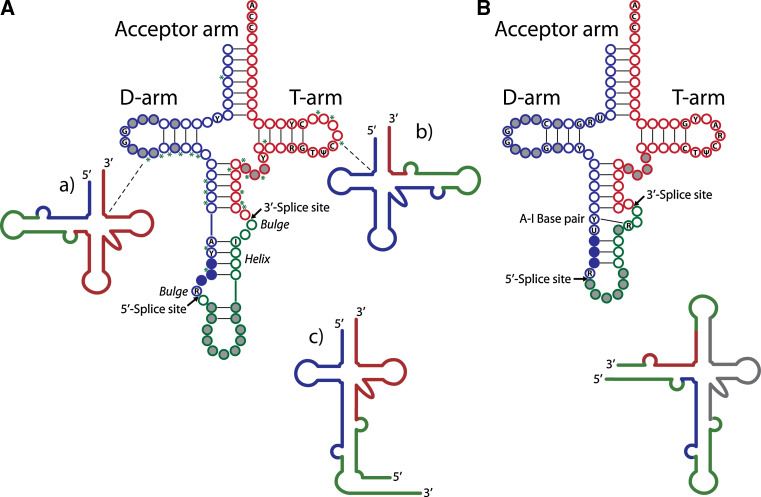
Position and conserved features of introns within end-matured archaeal and eukaryal pre-tRNAs [11]. **A** Secondary structure diagram of end-matured, intron-containing archaeal tRNAs. Schemes a and b represent end-matured tRNAs with introns in the D-arm or the T-arm, respectively. Scheme c depicts an end-matured split pre-tRNA assembled from two separate primary transcripts [25]. **B** Secondary structure diagram of end-matured, intron-containing eukaryotic pre-tRNAs. The accompanying scheme represents non-canonical introns and end processing sites of a permuted pre-tRNA transcript in the red alga *C. merolae*. The non-canonical intron in the acceptor arm is assumed to be excised by tRNA end processing enzymes rather than pre-tRNA splicing factors [19]. A, adenosine; C, cytosine; G, guanosine; U, uridine; Ψ, pseudouridine; Y, pyrimidine; R, purine; *asterisks* indicate positions of additional introns, 5′-exonic regions are depicted in *blue*, 3′-exonic regions in *red* and intronic regions in *green*. *Full grey circles* indicate nonconserved nucleotides in regions of variable length, *full blue circles* indicate the position of the anticodon

In archaea, as many as 70% of genetically encoded pre-tRNAs can be disrupted by introns of 16–44 nucleotides in length, which are characterized by the presence of a highly conserved “bulge-helix-bulge” motif [14, 15]. In some archaeal species, pre-tRNAs harbor up to three introns inserted at various sites [16] (Fig. 1A, schemes a and b). More recently, split tRNA (Fig. 1A, scheme c) and tri-split archaeal tRNA genes, which encode for parts of the mature domain, have been discovered [17, 18]. This type of tRNA arises from distinct transcription units that are joined by trans-splicing to yield a functional tRNA.

Eukaryal tRNA introns are relatively short and range from 12 to 104 nucleotides in e.g., humans [8]. In eukarya, tRNA introns are less abundant than in archaea—e.g., only 6% of human tRNA genes and 20% of yeast tRNA genes are disrupted by introns [8]—and do not display any conserved structural motifs. Rather, the position of the intron is highly invariable in almost all known eukaryotic pre-tRNA genes (Fig. 1B). An exception to this rule is exemplified by the non-canonical introns found in the circularly permuted tRNA genes of the red alga *Cyanidioschyzon merolae* (see scheme accompanying Fig. 1B) [19].

## Origin and function of introns within tRNA

Several hypotheses for evolutionary origins of tRNA introns have been put forward. An “intron-early” scenario proposes the existence of discontiguous primitive tRNA genes interrupted by introns harboring, for example, genes encoding splicing enzymes or aminoacyl transferases. Throughout evolution, these introns could have lost their initial functions and gradually acquired their current features [20]. The related “split early” hypothesis speculates that tRNA introns derive from flanking sequences of a priori split tRNA genes [21, 22]. The contrasting “intron late” or “split late” scenarios assume that tRNA introns arose later in evolution and propagated themselves by gene conversion or transposition [23]. Recent analyses of archaeal genome sequences suggest that tRNA introns in at least some archaeal species have been inserted into contiguous tRNA genes, e.g., by transposition [24]. A following separation of the sequences encoding the mature domains could have given rise to today’s split tRNA genes [25, 26].

As their phylogenetic origins, the functions of tRNA introns are still being explored. The presence of introns in some particular pre-tRNAs has been demonstrated to be required for enzymatic modification of nucleotides such as methylation [27] and pseudouridylation [28–30]. Only recently has the function of tRNA introns in vivo begun to be addressed in genetic experiments carried out with *Saccharomyces cerevisiae*. Here, removal of all introns of a particular tRNA isodecoder family did not affect growth or translation of the obtained mutants at laboratory conditions [31]. It is hoped that similar studies will yield further insights into the relevance of tRNA introns.

## Endonucleolytic excision of tRNA introns

**Fig. 2:**
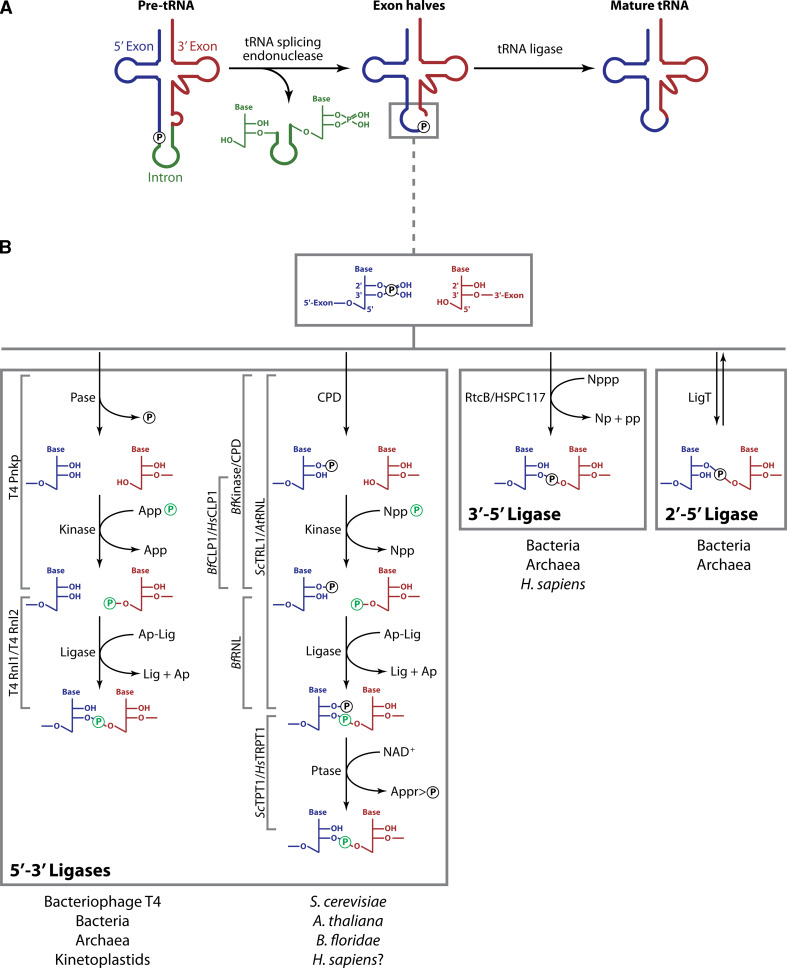
Enzymatic splicing of pre-tRNA. **A** Splicing of introns within pre-tRNA transcripts in archaea and eukaryotes is accomplished by separate cleavage and ligation reactions catalyzed by an endonuclease and a ligase enzyme, respectively. 5′-exons are depicted in *blue*, 3′-exons in *red*, and introns in *green*. **B** RNA ligation mechanisms. *Brackets in gray* indicate the names of enzymes catalyzing the indicated reactions. *Pase* phosphatase; *CPD* cyclic phosphodiesterase; *Appp* adenosine 5′-triphosphate; *App* adenosine 5′-diphosphate; *Ap* adenosine 5′-monophosphate; *Gppp* guanosine 5′-triphosphate; *Gpp* guanosine 5′-diphosphate; *Nppp* unspecified nucleoside 5′-triphosphate; *Np* unspecified nucleoside 5′-monophosphate; *pp* pyrophosphate; *Ap-Lig* adenylated ligase protein; *NT-domain* nucleotidyl transferase domain; *Ptase* 2′-phosphotransferase; *NAD* ^*+*^ nicotinamide adenine dinucleotide; *Appr>p* ADP-ribose-1″,2″- cyclic phosphate

Yeast mutants accumulating pre-tRNAs [32] initially provided access to substrates suitable for studying tRNA processing in biochemical experiments [33, 34]. These studies led to the conclusion that splicing of pre-tRNAs occurs in two steps. During an endonuclease reaction, the intron is first excised and the resulting tRNA exon halves are then ligated to form a mature tRNA (Fig. 2A) [35]. The biochemical fractionation of extracts catalyzing pre-tRNA cleavage has led to the identification of tRNA endonuclease proteins in archaea [36] and yeast [37], facilitating the characterization of the homologous human proteins [38].

In both archaea and eukarya, the tRNA endonuclease catalyses the cleavage of pre-tRNA at the two exon–intron boundaries and generates tRNA exon halves displaying a 2′,3′-cyclic phosphate at the 3′-end of the 5′-exon and a 5′-hydroxyl at the 5′-end of the 3′-exon (Fig. 2B, box at the top). In addition, the reaction yields a linear intron with 2′,3′-cyclic phosphate and 5′-hydroxyl termini (Fig. 2A). Although being mechanistically and evolutionarily related [39], it has been shown that archaeal and eukaryal tRNA splicing endonucleases differ with respect to substrate recognition [9].

### Archaeal tRNA endonucleases

Studies modifying the substrates used for in vitro processing reactions revealed that splice site recognition by archaeal tRNA splicing endonucleases relies on the presence of the bulge-helix-bulge motif [14]. Archaeal tRNA endonucleases consist of two dimers, each composed of a catalytic and a structural subunit. These dimers combine in an antiparallel fashion and interact with the pseudosymmetric substrate. Each exon–intron boundary is cleaved by one of the diagonally juxtaposed catalytic subunits, which together form the two composite active sites [40]. Despite the conservation of the recognition motif, four types of oligomeric organization of archaeal splicing endonucleases have co-evolved with minor changes of their substrates [41] giving rise to α_4_ (homotetramers, found in Euryarchaeota), α_2_ (homodimers, found in Euryarchaeota), (αβ)_2_ (dimers of heterodimers found in some Crenarchaeota such as e.g., *Nanoarchaeum equitans*), and αβγδ (heterotetramers, also found in Crenarchaeota) enzymes [39, 42–44]. In the case of the homotetrameric endonucleases, two of the four identical chains function as structural subunits whereas the two remaining polypeptides serve as catalytic subunits [45]. The monomers of the homodimeric splicing endonucleases might have arisen from an in-frame gene duplication and thus encode for a fusion of a structural and a catalytic domain [42].

### Eukaryal tRNA endonucleases

The eukaryal tRNA endonucleases are phylogenetically related to their archaeal counterparts [39]. The yeast endonuclease is of the αβγδ type and consists of two catalytic subunits, *Sc*SEN2 and *Sc*SEN34, and two structural subunits, *Sc*SEN15 and *Sc*SEN54 [37]. All four yeast subunits have homologous human proteins termed *Hs*TSEN2, *Hs*TSEN34, *Hs*TSEN15, and *Hs*TSEN54 [38]. Although it has been shown that at least one eukaryal tRNA endonuclease retained the ability to process archaeal pre-tRNA substrates [46], eukaryal endonucleases seem to have a different mode of splice site recognition: Eukaryal tRNA endonucleases cleave pre-tRNA substrates at a conserved distance from a structural feature located in the mature domain [47]. Apart from a strictly conserved A:I base pair (Fig. 1B) [48], little sequence constraint seems to exist for the intron itself, which can be extensively mutated without disturbing its proper recognition by the endonuclease [49].

## RNA ligation

Proteins catalyzing the final step of tRNA splicing—ligation of tRNA exon halves [35]—have first been identified by biochemical fractionation approaches in yeast [50, 51] and plants [52]. Initial biochemical experiments already indicated that the ligation step is mechanistically not as conserved among the different archaeal and eukaryal organisms as the endonuclease reaction [53–56]. Interestingly, although not requiring tRNA splicing enzymes, *Escherichia coli* has been shown to catalyze ligation of tRNA exon halves [57]. Even earlier, an RNA ligase activity had been identified in bacteriophage T4-infected *E. coli* [58]. These experiments led to the conclusion that, based on the type of phosphodiester bond established, distinct types of RNA ligase activity exist [10] (Fig. 2B). Bacteriophages on one hand, and fungi and plants on the other, utilize two related but mechanistically distinct multistep reactions to prepare the 2′,3′-cyclic phosphate and 5′-hydroxyl termini produced by the upstream endonuclease reaction for ligation (Fig. 2B, left box). Both mechanisms require the hydrolysis of the 2′,3′-cyclic phosphate and phosphorylation of the 5′-hydroxyl prior to ligation and as a result the phosphate at the newly formed phosphodiester linkage originates from the nucleotide triphosphate cofactor used for the kinase reaction [10]. To indicate that a 5′-phosphate is joined to a 3′-hydroxyl moiety, these two pathways are hereinafter referred to as 5′–3′ RNA ligase mechanisms. In addition, two mechanisms exist that incorporate the cleavage site-derived phosphate into the splice junction (Fig. 2B, central and right boxes). 3′–5′ RNA ligation converts 2′,3′-cyclic phosphate and 5′-hydroxyl termini into a 3′,5′ phosphodiester and can be detected in archaeal and vertebrate cell extracts. During 2′–5′ RNA ligation, predominantly found in eubacteria and archaea, 2′,3′-cyclic phosphate and 5′-hydroxyl moieties are joined to give rise to a 2′,5′-phosphodiester bond [59, 60].

### 5′–3′ RNA ligases in bacteriophages, bacteria, archaea, and kinetoplastids

Several bacterial species have evolved to sacrifice individual members of their populations upon phage infection by activating various suicide response pathways [61]. One of these suicide response mechanisms entails the activation of the latent anticodon nuclease PrrC in phage-infected *E. coli* CTr5x. The nuclease—kept in an inactive state in absence of phage infection—cleaves the host’s tRNA^Lys^ in the anticodon loop to shut down protein synthesis and thus impair phage propagation. Cleavage by PrrC yields 2′,3′-cyclic phosphate and 5′-hydroxyl termini resembling the products of tRNA splicing endonuclease reactions. Bacteriophage T4, however, has evolved to cope with this defense mechanism. The phage *pnk* and *rli* genes encode for proteins capable of restoring cleaved tRNA^Lys^ [62]. It has been demonstrated that T4 Pnkp (the protein encoded by the *pnk* gene) acts both as phosphatase [63] and polynucleotide kinase [64] thus converting the 2′,3′-cyclic phosphate and 5′-hydroxyl of tRNA halves into 2′,3′-*cis* diol and 5′-phosphate termini (Fig. 2B, left box, left branch). T4 Rnl1 (encoded by the *rli* gene) subsequently joins these tRNA halves by 5′–3′ RNA ligation thus restores a functional tRNA [65].

**Fig. 3:**
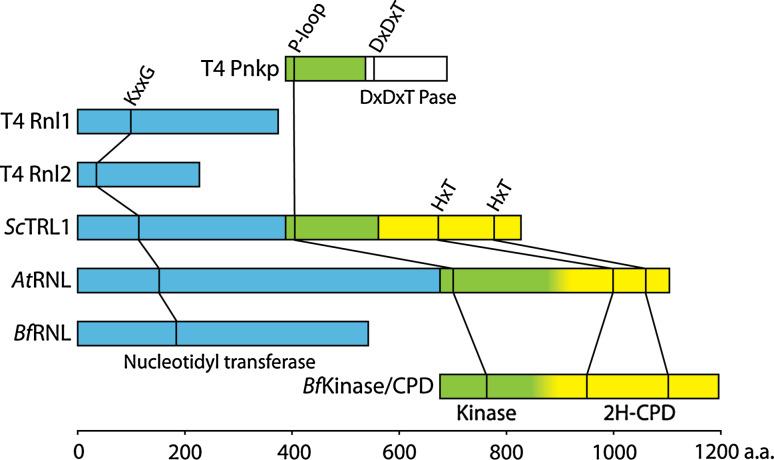
Domain and polypeptide organization of archetypical RNA ligase systems from bacteriophage T4 (T4 Pnkp, T4 Rnl2, and T4 Rnl1) [70, 77, 79, 80], *S. cerevisiae* (*Sc*TRL1) [74], *A. thaliana* (*At*RNL) [52, 75], and *B. floridae* (*Bf*RNL and *Bf*Kinase/CPD) [87]. Nucleotidyl transferase domains are depicted in *light blue*, kinase domains in *green*, the DxDxT Pase (aspartic acid-based phosphatase) domain in *white* and 2H-CPD (two conserved histidine based phosphoesterase) domains in *yellow*. No published domain boundaries are currently available for the *A. thaliana* and *B. floridae* Kinase/CPD modules, which is indicated by a gradual transition between the two respective colors. The positions of key motifs are drawn to scale. *Pnkp* polynucleotide kinase/phosphatase; *Pase* phosphatase; *CPD* cyclic phosphodiesterase

In addition to T4 Rnl1, another RNA ligase, T4 Rnl2, has been identified in the genome of bacteriophage T4 [66]. T4 Rnl1 and T4 Rnl2 constitute distinct and only distantly related protein families sharing characteristic features (Fig. 3). Members of the Rnl2 family have been detected and examined in viral, bacterial, and archaeal genomes, suggesting a common phylogenetic origin and possibly a function in RNA repair [66–70]. Potentially, this type of ligases might also be involved in archaeal tRNA splicing. In trypanosomatids, 5′–3′ RNA ligases of the Rnl2 type are involved in RNA-guided editing of mitochondrial pre-mRNAs by nucleolytic cleavage and ligation [71].

### 5′–3′ RNA ligases in yeast and plants

The finding that tRNA splicing in yeast occurs as a two-step reaction, an ATP-independent endonuclease reaction, and an ATP-dependent ligase reaction constituted the first piece of evidence for the existence of a eukaryotic tRNA ligase [35]. Later, an RNA ligase activity was characterized in wheat germ extracts [55, 72] by using a nucleolytic fragment—obtained by RNase T1 digestion of tobacco mosaic virus RNA [73]—as an artificial ligase substrate. A thorough characterization of the splice junction revealed that a 2′-phosphomonoester-3′,5′-phosphodiester linkage is established in this system [55, 72]. The same has been shown to be true for the yeast tRNA ligase [50]. Yeast (*Sc*TRL1) and plant (exemplified by *At*RNL from *Arabidopsis thaliana*) tRNA ligases—both identified by activity-guided chromatographic purification [50–52]—are multifunctional enzymes harboring all the activities required to join the 2′,3′-cyclic phosphate and 5′-hydroxyl termini in three functionally independent domains [74, 75] (Fig. 3). First, the 2′,3′-cyclic phosphate is hydrolyzed by a cyclic phosphodiesterase (CPD) activity to yield a 2′-phosphate-3′-hydroxyl terminated 5′-exon [50, 76]. In a second step, the 5′-hydroxyl at the 3′-exon is phosphorylated by a kinase activity [50]. The actual ligation is preceded by ATP-dependent adenylation of a lysine located within a KxxG consensus motif in the active site of the nucleotidyl transferase domain [50, 76]. Next, the enzyme transfers the adenyl moiety to the 5′-phosphate at the 3′-exon [50]. The adenylated RNA is then joined to the 2′-phosphate-3′-hydroxyl group at the 5′-exon with the concomitant release of AMP [51, 52] (Fig. 2B, left box, right branch and Fig. 3). Although RNA ligases of bacteriophage T4, fungi, and plants share important mechanistic features and key residues required for catalysis (Fig. 2B, left box and Fig. 3), their overall sequence similarity is low [52]. However, the presence of the conserved nucleotidyl transferase domain with its ligase motifs in bacteriophage T4, fungal and plant RNA ligases suggests a common evolutionary origin for these enzymes [69]. Despite their relatedness, several differences exist between yeast, plant, and bacteriophage T4 ligases. For example, ligase- and kinase/phosphatase activities reside on separate polypeptides in bacteriophage T4 whereas yeast and plant tRNA ligases are multifunctional proteins harboring all activities required for ligation (Fig. 2B, left box, right branch and Fig. 3). Hydrolysis of 2′,3′-cyclic phosphates by yeast and plant CPD domains yields 2′-phosphate-3′-hydroxyl products rather than the 2′,3′-*cis* diol termini generated by T4 Pnkp (Fig. 2B, left box) [50, 55, 72, 76]. This arises from the distinct phosphoesterase domains employed by the bacteriophage T4 (aspartic acid based DxDxT phosphoesterase domain [77]) and yeast/plant (2H phosphoesterase domain, characterized by two conserved histidines [78]) ligation pathways [77, 79, 80] (Fig. 3). In contrast to bacteriophage T4 ligases, the yeast and plant enzymes do not accept 3′-phosphate RNA substrates and ligation typically depends on the presence of a 2′-phosphate at the substrate termini [79, 81]. Yeast and plant RNA ligases generate splice junctions bearing a 2′-phosphate [72]. To convert this non-canonical 2′-phosphomonoester-3′,5′-phosphodiester linkage into a 3′,5′-phosphodiester, an NAD^+^-dependent phosphotransferase, termed *Sc*TPT1 in yeast, removes the 2′-phosphate generating ADP-ribose-1″,2″-cyclic phosphate (Fig. 2B, left box, right branch) [82–85]. Yeast and plant tRNA ligases also subtly differ with respect to substrate selection. While yeast tRNA ligase prefers tRNA exon halves over artificial substrates such as, e.g., linear introns [51], the plant enzyme ligates circular introns as efficiently as tRNA exon halves in direct competition experiments [52].

### 5′–3′ RNA ligases in higher eukaryotes

Although a 5′–3′ RNA ligase activity has been detected in biochemical experiments carried out with HeLa cell extracts [86], no homologues of known 5′–3′ RNA ligase proteins could be identified in animals [52]. It therefore seems likely that the 5′–3′ RNA ligase in animals has diverged too far from the RNA ligases already known in order to be identified by existing algorithms or represents a completely novel type of enzyme. This view is supported by the recent identification of a 5′–3′ RNA ligase in the cephalochordate *Branchiostoma floridae* (*Bf*RNL) featuring a stand-alone ligase protein with weak homology to yeast and plant RNA ligases [87]. In this system, the cyclic phosphodiesterase and kinase modules (*Bf*Kinase/CPD or *Bf*CLP1) do not reside on the ligase polypeptide (Fig. 2B, left box, right branch and Fig. 3). Enzymes catalyzing kinase (*Hs*CLP1) [88], cyclic phosphodiesterase (*Hs*CNP) [89–91], and phosphotransferase (*Hs*TRPT1) [92] activities have been identified in humans. The fact that these enzymes can function in tRNA splicing pathways in vivo [93–95] has triggered speculation that a yet unidentified 5′–3′ RNA ligase protein might also exist in humans [87]. In support of this assumption, the RNA kinase, *Hs*CLP1, is an integral component of the tRNA endonuclease complex, suggesting the occurrence of coupled endonuclease and kinase reactions in humans [38, 88]. In contrast, tRNA exon half phosphorylation is associated with ligation in *S. cerevisiae*, where ligase and kinase activities both reside on *Sc*TRL1. However, the relevance of the 5′–3′ RNA ligase mechanism for tRNA splicing in vertebrates is under debate since the 2′-phosphotransferase is not essential in mice [96].

To account for the substantial differences in sequence and domain organization, it has been suggested to group 5′–3′ RNA ligases in bacteriophages, yeast, plants, and animals into classes [66, 87]; however, no uniform nomenclature has become generally accepted to date.

### 3′–5′ RNA ligases in bacteria, archaea, and animals

Biochemical studies revealed that ligation of tRNA exon halves in vertebrates and archaea is mainly achieved by an alternative mechanism. A 3′–5′ RNA ligase activity resulting in incorporation of the precursor-derived 2′,3′-cyclic phosphate into the splice junction as a 3′,5′-phosphodiester was for the first time detected in HeLa cell extracts (Fig. 2B, central box) [53, 56, 97]. The same reaction has later been shown to occur in archaeal cell extracts [54, 98, 99]. Intensive attempts to identify the human tRNA ligase proteins by chromatographic purification were triggered by these initial findings [100, 101]. Recently, chromatographic purification led to the identification of RtcB/HSPC117 proteins as 3′–5′ RNA ligases in the crenarchaeon *Pyrobaculum aerophilum* and in humans [102, 103]. Concurrently, a candidate approach revealed RtcB in *Escherichia coli* as a 3′–5′ RNA ligase [104]. The high degree of conservation of HSPC117/RtcB proteins suggested a shared role for this protein family in many organisms already during their initial characterization [102–104].

#### Archaeal and bacterial RtcB proteins

RNA ligase activity of the recombinant RtcB proteins from *P. aerophilum* and *E. coli* strictly depends on the presence of bivalent metal ions [102, 104]. Studies probing the active sites of archaeal and bacterial RtcB by mutagenesis [102, 104, 105] could in part confirm early predictions concerning essential residues based on structural analyses of homologous RtcB proteins (pdb files 1UC2 and 2EPG) [106, 107]. However, all crystal structures of RtcB proteins available to date represent *apo* forms of the enzymes and thus the exact functions of individual amino acids lining the presumed active site cannot be assigned unambiguously. Extended structural studies of RtcB proteins in complex with bivalent metal ions, additional cofactors, and RNA substrates will be required to clarify the active site geometry and the distinct metal ion specificity observed for enzymes from different species [102, 104].

Initially, HSPC117/RtcB proteins were assumed to catalyze the direct nucleophilic attack of the 2′,3′-cyclic phosphate by a 5′-hydroxyl group, which seemed likely for two main reasons. First, 2′,3′-cyclic phosphates are energy-rich substrates with a favorable leaving group [108] and second, HSPC117/RtcB-catalyzed ligation reactions did not seem to be strictly dependent on the addition of nucleotide triphosphate cofactors [102–104, 108]. However, only enzyme preparations that were rigorously purified in the presence of chelating agents proved to yield RtcB preparations sufficiently pure to demonstrate the real cofactor requirements of RtcB-catalyzed 3′–5′ RNA ligation [108]. Thus, 3′–5′ RNA ligation by RtcB proteins occurs as a sequential reaction involving the stoichiometric hydrolysis of nucleotide triphosphates rather than direct nucleophilic attack of the 2′,3′-cyclic phosphate by a 5′-hydroxyl group [108]. One advantage of this somewhat counterintuitive strategy might lie in the suppression of the backward reaction—the cleavage of the newly formed phosphodiester—as it is catalyzed by bacterial 2′–5′-ligases (see below) [59].

#### The human tRNA ligase complex

**Fig. 4:**
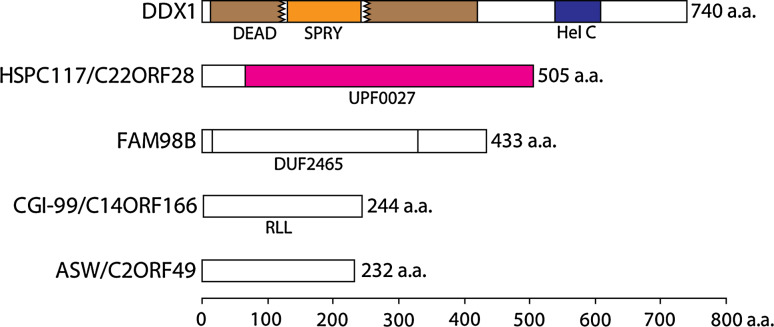
Domain organization of HSPC117 complex components (listed together with frequently encountered synonyms). Pfam domains are abbreviated as follows:* DEAD* DEAD/DEAH box helicase domain;* SPRY* SPRY domain (unknown function);* Hel C* Helicase conserved C-terminal domain;* UPF0027* Domain of unknown function;* DUF2465* Domain of unknown function;* RLL* Putative carnitine deficiency-associated protein domain

3′–5′ RNA ligation appears to be the prevalent human tRNA splicing pathway [53, 56] and relies on HSPC117 as the essential ligase component as supported by two experimental observations: RNAi-mediated depletion of HSPC117 severely impairs tRNA maturation and mutation of a strictly conserved cysteine residue abolishes ligase activity of the affinity purified protein [103]. Human HSPC117, together with the proteins DDX1, CGI-99, FAM98B, and ASW, forms a stable complex of about 200 kDa (Fig. 4) [103]. Even earlier, the observed co-selection of HSPC117, DDX1, and CGI-99 with cruciform DNA duplexes hinted that these three proteins might interact [109]. In contrast to depletion of HSPC117, RNAi-mediated silencing of the associated proteins does not severely affect RNA ligase activity in HeLa cell extracts, suggesting further functions of the interacting proteins that remain to be explored [103]. Potential roles of these accessory proteins may include targeting of the complex to appropriate cellular compartments, stabilization of the essential subunit, interaction with substrates or adaptor proteins and prevention of illegitimate, promiscuous ligation. Moreover, individual complex components may mediate a transient association of the ligase complex with the tRNA endonuclease, as it has been suggested for the yeast tRNA endonuclease and ligase [110]. In contrast to its archaeal and bacterial orthologues, recombinant HSPC117 did not act as an RNA ligase in vitro. In addition, HSPC117 per se was incapable of replacing the yeast tRNA ligase *TRL1* in complementation experiments (J. Popow, unpublished results).

The human tRNA ligase complex components seem to be constitutively and widely expressed in all human and mouse tissues analyzed so far, indicating that these proteins cooperate in a great variety of cellular contexts [103, 111, 112]. DDX1 is a member of the DEAD-box family of putative RNA helicases characterized by the presence of nine conserved sequence motifs [113]. The DEAD-box helicase domain of DDX1 is interrupted by the insertion of a SPRY domain (Fig. 4) detectable in numerous proteins and presumably acting as a protein interaction platform [114, 115]. DDX1 has been associated with many molecular functions ranging from mRNA processing [116, 117] to recognition of DNA double-strand breaks [118] and has been demonstrated to exhibit 3′–5′ RNA unwinding activity [117]. The association of DDX1 with HSPC117 suggests an involvement in tRNA splicing. On the other hand, RNAi-mediated depletion of DDX1 only mildly impaired tRNA maturation activity, suggesting that its function might be dispensable for RNA ligation by HSPC117 in HeLa cell extracts [103]. Alternatively, a lack of DDX1 might also have been compensated for by other proteins present in the assayed extracts. Further experiments assessing the effect of mutations within conserved motifs of DDX1 on ligase activity—carried out with purified RNA ligase complex—might successfully address the function of this protein in tRNA splicing. Co-localization studies and extended analysis of proteins associating with DDX1 could answer the question whether DDX1 mediates its functions in conjunction with HSPC117 or whether it is a shared component of multiple protein complexes acting in distinct biological processes. Furthermore, it might be rewarding to test whether the amplification of *DDX1* observed in cancerous cell lines [111, 119, 120], its function in viral replication [121–123], and dsRNA recognition in dendritic cells [124] are linked to RNA ligase activity or tRNA processing.

Published information concerning the function of CGI-99 is scarce. Although the protein has been found to interact with human [112, 125] and viral [125] proteins in yeast two-hybrid assays, these studies provided little insight into its molecular function. It remains to be determined whether the interaction of CGI-99 with the PA subunit of influenza virus polymerase [125] or its modulation of transcription by RNA polymerase II [126] is in any respect related to RNA ligase activity.

The identification of FAM98B as a human tRNA ligase complex component is the first piece of information published concerning its molecular function. Within the context of tRNA splicing, the exact role of FAM98B needs to be established. Since its RNAi-mediated depletion has almost no impact on RNA ligase activity in HeLa cell extracts [103], it is likely that FAM98B acts as an interaction platform recruiting accessory proteins or RNA substrates. On the other hand, it might also be the case that other cellular proteins compensated for a lack of FAM98B in these experiments. Alternatively, FAM98B may mediate completely unrelated, yet-to-be-discovered functions of HSPC117 complexes.

**Fig. 5:**
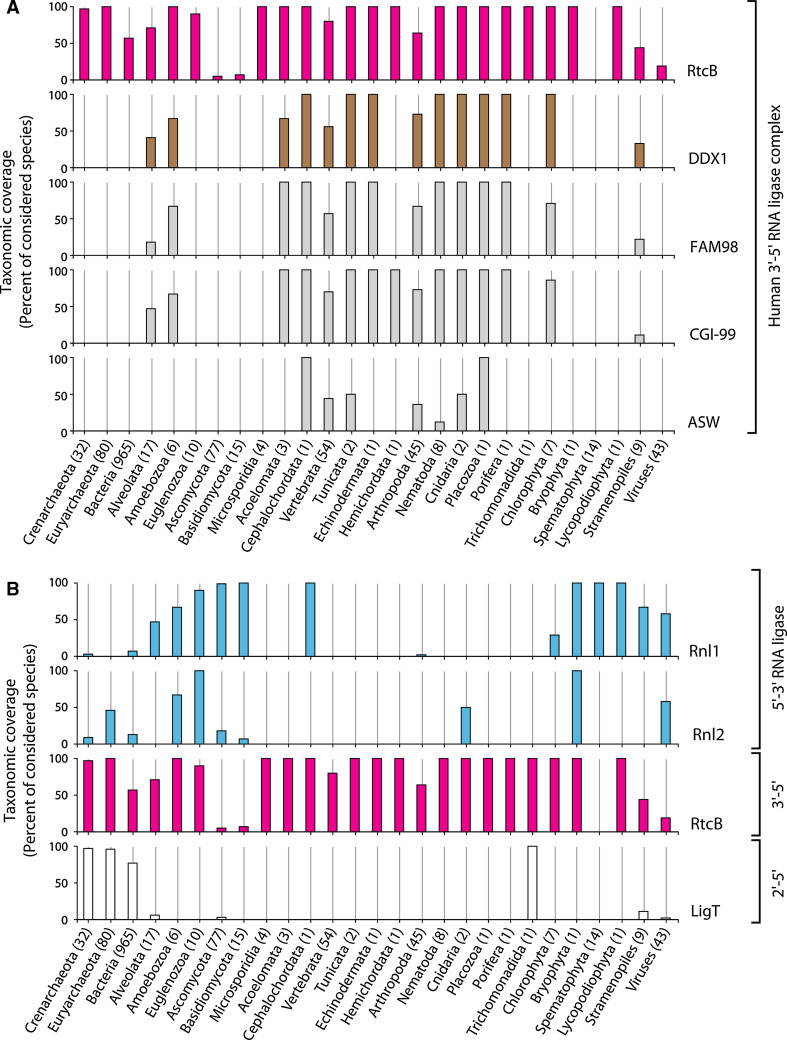
Phyletic distribution of **A** HSPC117-associated proteins and **B** RNA ligase enzymes. *Numbers in brackets* next to category axis labels indicate the total amount of species in the respective taxonomic group and *bars* represent the percentage of species with detectable homologues. Homologues of proteins (*right-hand side* labels) were identified within the NCBI non-redundant protein database by BLAST or Hidden Markov Model searches (see supplemental material for further details)

ASW has first been characterized in a genetic screen searching for genes differentially expressed in early neural specification in *Xenopus laevis* [127]. Although this study could demonstrate that alteration of ASW levels in *X. laevis* leads to severe developmental defects, it provided limited insights into mechanistic aspects of ASW function. ASW has been speculated to be specific for vertebrates as no homologous genes were detectable in *Drosophila melanogaster* or *Caenorhabditis elegans* [127]. Nonetheless, homologues of ASW are detectable in the genomes of at least some arthropods and nematodes (Fig. 5A). Future studies might answer the question of whether the link between ASW and HSPC117 explains any of the developmental defects observed upon manipulation of ASW levels in *X. laevis*.

Taken together, apart from HSPC117, which appears to be conserved among all domains of life, the additional components of the HSPC117 complex do not show a comparably broad taxonomic coverage. Orthologues of DDX1, FAM98B, and CGI-99 are spread more widely than ASW, which seems to be restricted to fewer species (Fig. 5A).

### 2′–5′ RNA ligases in archaea and bacteria

**Fig. 6:**
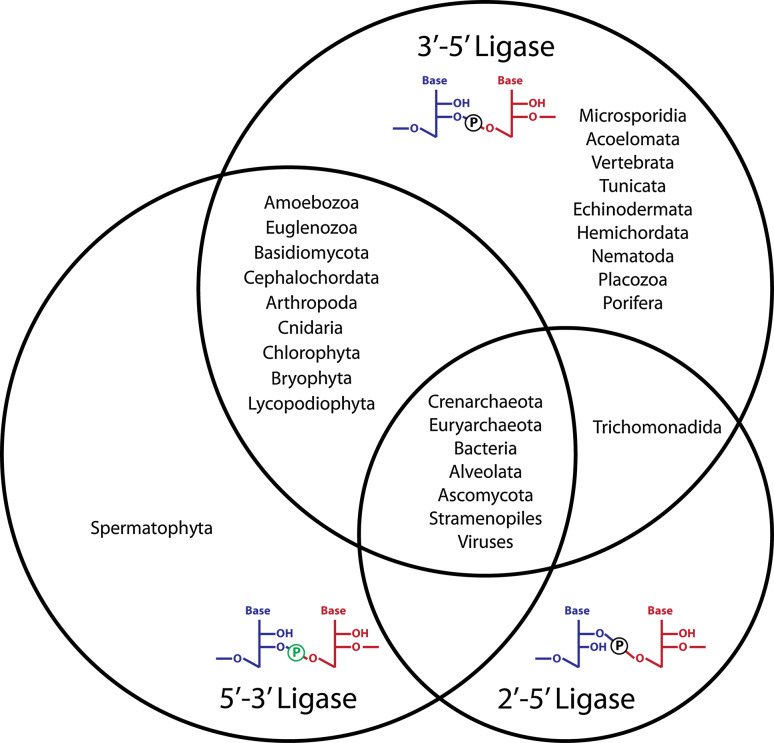
Schematic illustration of the phyletic distribution of identified RNA ligase polypeptide sequences

Another RNA ligase activity leading to the unusual 2′,5′-phosphodiester bond has been shown to act in extracts prepared from various bacterial species (Fig. 2B, right box) [57]. Its biochemical purification led to the identification of the ligase gene *ligT*, conserved not only in bacteria but also in euryarchaeota and crenarchaeota [59, 78]. The apparent dependence of its activity on the presence of modifications in its tRNA substrates suggests that it is involved in repair or processing of tRNA in its host [10, 59]. LigT from *E. coli* catalyses an ATP-independent equilibrium reaction between 2′,3′-cyclic phosphate and 5′-hydroxyl termini and 2′,5′-phosphodiester bonds [59]. More recently, a homologous archaeal 2′–5′ RNA ligase has been characterized in the euryarchaeon *Pyrococcus furiosus* confirming the broad phyletic occurrence of LigT proteins [60]. Although the high degree of conservation suggests an evolutionarily important role for 2′–5′ ligase genes, the exact biological function of LigT proteins and 2′,5′-phosphodiester bond formation are still unknown [59, 60, 78]. Interestingly, a few eukaryotic genomes also encode homologs of bacterial and archaeal LigT proteins (Figs. 5B, 6). The activities and biological functions of these proteins have not yet been addressed.

### Phyletic association of RNA ligases

RNA ligase proteins are detectable within all major lines of descent. Considering the different ligation mechanisms and classes of RNA ligase polypeptides, it seems, however, that several species abandoned one or the other mechanism of RNA ligation (Figs. 5B, 6). Most representatives of the Basidiomycota and Spermatophyta lineages might have lost RtcB/HSPC117 homologues during the course of evolution. However, there seem to be rare instances (such as the basidiomycote *Piriformospora indica*, see supplemental information for details) where an RtcB/HSPC117 protein has been retained [52, 102, 103, 128] (Figs. 5B, 6). None of the 5′–3′ RNA ligases known to date could be allocated to several higher eukaryotes, such as e.g., humans (Fig. 5B). Nevertheless, this picture might change upon the identification of a currently unknown type of 5′–3′ RNA ligase. The detection of 5′–3′ RNA ligase activity in humans [86] together with the recent discovery of the *B. floridae* 5′–3′ RNA ligase *Bf*RNL [87] argue in favor of this view.

## Subcellular localization of tRNA splicing in eukaryotes

The subcellular localization of tRNA processing events does not seem to be conserved among all eukaryotes. Many localization studies were carried out in the yeast *S. cerevisiae* which, however, seems to differ in this respect from many other eukaryotes [129]. tRNA splicing is assumed to occur in the cytoplasm in *S. cerevisiae*, based on the localization of yeast tRNA endonuclease subunits to the outer mitochondrial membrane [130]. Controversial data have been reported concerning the localization of tRNA splicing in plants. On the one hand, some plant tRNA splicing enzymes at least partly localize to the nucleus [131]. On the other hand, impaired nuclear export of tRNA results in accumulation of unspliced pre-tRNA in *Arabidopsis* *thaliana* [132] arguing in favor of cytoplasmic tRNA splicing in plants in analogy to the coupling of nuclear export and cytoplasmic tRNA splicing in yeast *rna1*-*1* mutants [32, 133, 134]. In the vertebrate *X. laevis*, tRNA splicing—preceded by 3′-end formation including CCA addition—has been shown to occur in the nucleus by micro-dissection and micro-injection studies carried out with oocytes [135–137]. In accord with these observations, subunits of the human tRNA endonuclease have been reported to be nuclear proteins [38].

## Functions of RNA ligases unrelated to tRNA splicing

The biochemical characterization of identified tRNA ligase enzymes revealed that they accept not only tRNA exon halves but also a broad range of artificial substrates such as nucleolytic RNA fragments, synthetic RNA duplexes, and linear introns generated by tRNA endonucleases [52, 53, 55, 97, 102–105]. In addition, human cell extracts have been shown to not only ligate 2′,3′-cyclic phosphate bearing but also 3′-phosphate-terminated RNA substrates [97, 138]. Biochemical fractionation of HeLa extracts revealed that RNA 3′-phosphate terminal cyclase RTCD1 can convert RNA 3′-phosphates into 2′,3′-cyclic phosphates in an ATP-dependent reaction [139, 140]. Despite the thorough characterization of bacterial and human RNA 3′-phosphate terminal cyclase proteins [140, 141], the physiological function of this enzyme class is still unknown. The recent mechanistic characterization of RtcB from *E. coli* suggests that 3′-phosphate-terminated RNA is per se a substrate for this enzyme [108], raising questions about the genuine function of RNA terminal cyclase RtcA in *E. coli*. The potential to prepare a variety of RNA termini for ligation and the relaxed substrate specificity of the ligase itself in many organisms suggests that tRNA splicing enzymes might also act in other biological contexts. As an example of such a case, *Sc*TRL1 has been shown to be involved in stress-induced, non-canonical splicing of the *HAC1* transcript in *S. cerevisiae* [142, 143]. A related pathway acts in human cells, however, the involved RNA ligase has to date not been identified [144–147]. Furthermore, RNA ligases have been proposed as host factors for the propagation of viruses, viroids, and viroid-like satellite RNAs in humans and plants [148]. In fact, HSPC117 and DDX1 are involved in RNA processing during replication of the hepatitis delta virus [121], presumably acting as the host RNA ligase factors assumed to be required for cyclization of the viral RNA genome as previously proposed [149]. Furthermore, components of the HSPC117-complex together with the RNA cyclase, RTCD1, have been shown to interact with kinesin-associated RNA transport granules in mouse brain extracts [150]. The exact functions of these RNA metabolic enzymes in the context of RNA transport await further experiments. In addition to processing of RNA transcripts of various origins, RNA ligases are assumed to be involved in RNA repair pathways. Apart from the well-studied example of tRNA repair by bacteriophage T4 RNA ligase [62], further examples of RNA repair have been revealed [67, 79, 151]. These studies showed an amazing potential of RNA ligases to function as safeguards against the deleterious effects of cytotoxic nucleases in yeast and bacteria. Similar efforts might unveil examples of RNA repair in unanticipated physiological contexts not only in bacteria and yeast but also in higher organisms. The high degree of conservation of HSPC117/RtcB proteins and preliminary data indicating that HSPC117 seems to be encoded by an essential gene in mice [152] are highly suggestive of universal and important roles for this protein family. Assigning a biological function to the operon harboring the ligase, *rtcB*, and the cyclase, *rtcA*, in some bacteria may help to uncover functions of this type of ligase unrelated to tRNA splicing since *E. coli* does not encode for intron harboring tRNAs. Finally, the identification of genuine substrates interacting with RNA ligase proteins in vivo may yield fascinating insights into the various processes potentially requiring RNA ligases.

### Electronic supplementary material

Below is the link to the electronic supplementary material.

Supplementary material 1 (DOC 33 kb)
