# Supplementary material for: Diversity and roles of (t)RNA ligases
Source: Cell Mol Life Sci. 2012 Mar 17;69(16):2657–70. doi: 10.1007/s00018-012-0944-2 (PMC3400036; doi:10.1007/s00018-012-0944-2)
Supplement: Supplementary file 1 — Supplementary material 1 (DOC 33 kb) [file 18_2012_944_MOESM1_ESM.doc]

**Supplemental methods:**

Homology searches and taxonomic classification

The RtcB, FAM98, CGI-99, Rnl2 and LigT protein families were collected using HMMER v3 (default inclusion threshold 0.001) [1] with the following PFAM models [2]: UPF0027 (RtcB), DUF2465 (Fam98), RLL (CGI-99), RNA_ligase (Rnl2), and 2_5_RNA_ligase (LigT). ASW homologues were identified with NCBI PSI-BLAST applying significant E-values (<0.001), using human ASW as query (gi|13129094|) [3]. The human DDX1 protein (gi|119621300|) consists of three domains: an amino-terminal DEAD domain (P-loop NTPase superfamily) with a SPRY domain inserted, and the HelC domain (c-terminal helicase specific domain). We created a hidden Markov model (HMM) covering all three domains and applied highly significant e-values (<1e-38) in the following HMMER (v2) search to restrict to DDX1 specific helicases. The Rnl1 family was mainly collected with the PFAM model RNA_lig_T4. Two related RNA ligase families, one represented by the *Arabidopsis thaliana* protein *At*RNL (gi|145335250|) and the other one by the *Branchiostoma floridae* protein *Bf*RNL (gi|260818956|), were missing in the initial RNA_lig_T4 domain search. These families were identified with NCBI BLAST and HMM searches using own models (e-values <1e-3). To classify the Rnl1 family according to their phyletic distribution, the RNA_lig_T4 domain search was combined with the results of the two related families.

All searches were performed within the NCBI non redundant protein database; and the taxonomy was assigned with the NCBI taxonomy database [4]. For Figure 6, we reduced the organism names to the genus and species level, to avoid over-representation of taxonomic groups with high number of isolates (see Supplementary table 1 for a full listing of organism).

**Supplementary table 1:**

Phyletic distribution of RNA ligase proteins. NCBI protein gi IDs indicate the presence in the respective organism. No redundancy was removed on the protein level, therefore multiple gi IDs may represent a single protein.

**Supplementary references:**

[1] Johnson, L.S., Eddy, S.R. and Portugaly, E. (2010). Hidden Markov model speed heuristic and iterative HMM search procedure. BMC Bioinformatics 11, 431.

[2] Finn, R.D. et al. (2010). The Pfam protein families database. Nucleic Acids Res 38, D211-22.

[3] Altschul, S.F., Madden, T.L., Schaffer, A.A., Zhang, J., Zhang, Z., Miller, W. and Lipman, D.J. (1997). Gapped BLAST and PSI-BLAST: a new generation of protein database search programs. Nucleic Acids Res 25, 3389-402.

[4] Sayers, E.W. et al. (2011). Database resources of the National Center for Biotechnology Information. Nucleic Acids Res 39, D38-51.
